# Total Exenteration En Bloc with a Nephrectomy for Locally Advanced Cervical Cancer Invading a Pelvic Kidney—A Case Report and Literature Review

**DOI:** 10.3390/medicina56010033

**Published:** 2020-01-15

**Authors:** Nicolae Bacalbasa, Irina Balescu, Mihaela Vilcu, Simona Dima, Camelia Diaconu, Laura Iliescu, Alexandru Filipescu, Iulian Brezean

**Affiliations:** 1 “Carol Davila” University of Medicine and Pharmacy, 020021 Bucharest, Romania; nicolae_bacalbasa@yahoo.ro (N.B.); mihaela.vilcu@gmail.ro (M.V.); drcameliadiaconu@gmail.com (C.D.); laurailiescu@gmail.ro (L.I.); iulianbrezean@gmail.ro (I.B.); 2 Department of Obstetrics and Gynecology, “I. Cantacuzino” Clinical Hospital, 030167 Bucharest, Romania; 3 Department of Visceral Surgery, “Fundeni” Clinical Institute, 022328 Bucharest, Romania; simonadima@gmail.ro; 4 Department of Surgery, “Ponderas” Academic Hospital, 021188 Bucharest, Romania; 5 Department of Visceral Surgery, “I. Cantacuzino” Clinical Hospital, 030167 Bucharest, Romania; 6 Department of Internal Medicine, Clinical Emergency Hospital of Bucharest, 105402 Bucharest, Romania; 7 Department of Internal Medicine, “Fundeni” Clinical Institute, 022328 Bucharest, Romania; 8 Department of Obstetrics and Gynecology, “Elias” Emergency University Hospital, 011461 Bucharest, Romania; alexandrufilipescu@gmail.ro

**Keywords:** ectopic kidney, locally advanced cervical cancer, nephrectomy

## Abstract

*Introduction*: Extended pelvic resection might be the option of choice in patients presenting locally advanced cervical cancer. However, the possibility of a co-existence of an ectopic, pelvic kidney that is invaded by such a tumor is extremely rare. *Case Presentation*: A 54-year-old female patient, diagnosed with locally advanced cervical cancer in the presence of a pelvic kidney, was submitted to surgery with curative intent. A large, abscessed cervical tumor invading the urinary bladder and the rectum was found, so a total exenteration was planned. Intraoperatively, tumor invasion of the left kidney, which was found in an ectopic, pelvic position was also encountered; therefore, total pelvic exenteration in association with a left nephrectomy was successfully performed. *Conclusions:* The presence of an ectopic, pelvic disposition of the kidney makes it susceptible to be invaded by locally advanced pelvic tumors; in such cases, a nephrectomy might also be needed.

## 1. Introduction

An ectopic kidney refers to the situation in which developmental arrest of the renal ascent is encountered, leading to the location of this viscus in the pelvic, iliac or abdominal area [1]. Most often, a pelvic kidney is situated opposite to the sacrum bone and below the aortic bifurcation and presents an incomplete rotation [2]. Such cases also present certain modifications with regard to the length and disposition of the ureter and vascular supply: In up to half of these patients, a certain degree of hydronephrosis can be encountered, while the renal arteries can arise from the distal aorta, aortic bifurcation, common or external iliac arteries or even from the inferior mesenteric artery [1,2]. Therefore, its pelvic location makes it susceptible to be invaded by all the tumoral processes of pelvic origin, while the surgical approach can be significantly influenced. The incidence of this anatomical particularity is estimated to be one in 2100 to 3000 cases [3].

## 2. Case Presentation

**Figure 1:**
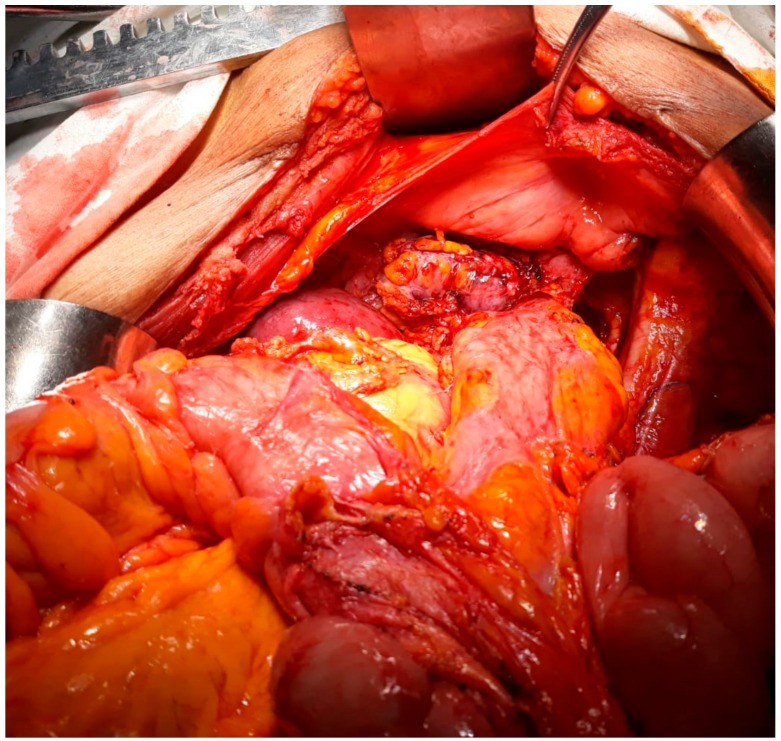
Initial intraoperative aspect: Large pelvic tumor invading the left kidney with a pelvic location.

**Figure 2:**
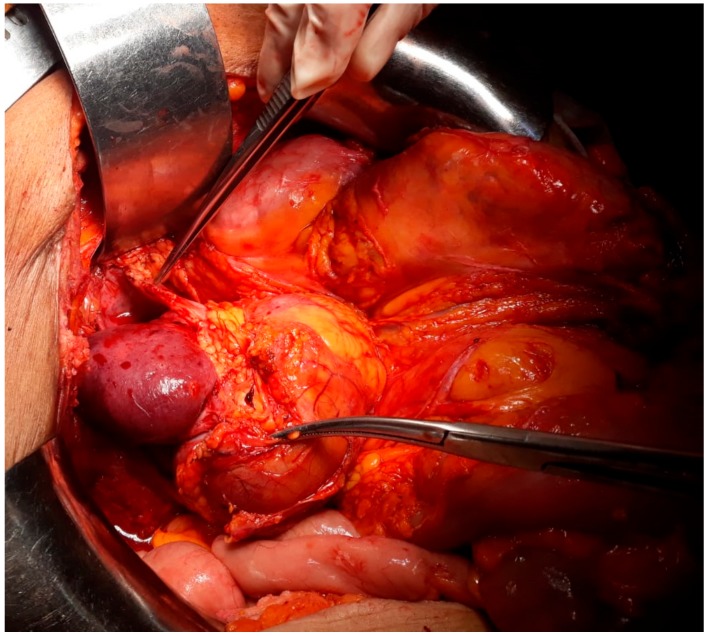
The aspect after tumoral mobilization—presence of ureteral invasion as well as renal invasion.

**Figure 3:**
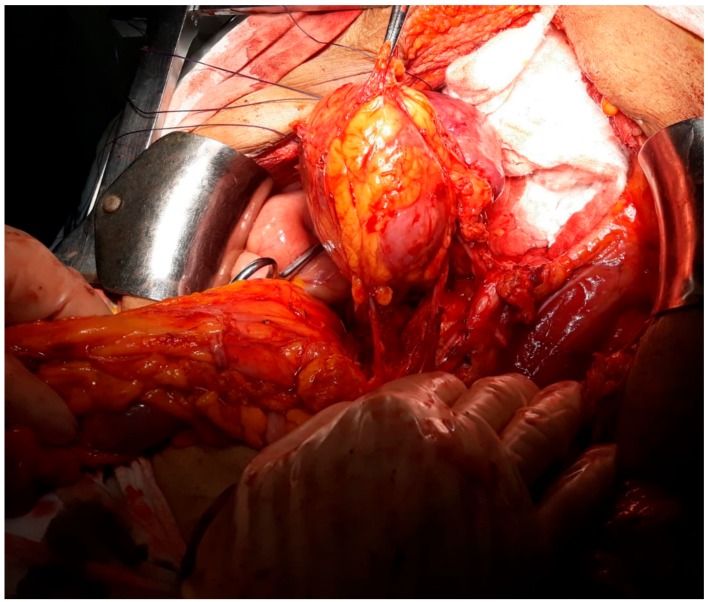
The aspect after rectal sectioning and posterior dissection of the tumor.

**Figure 4:**
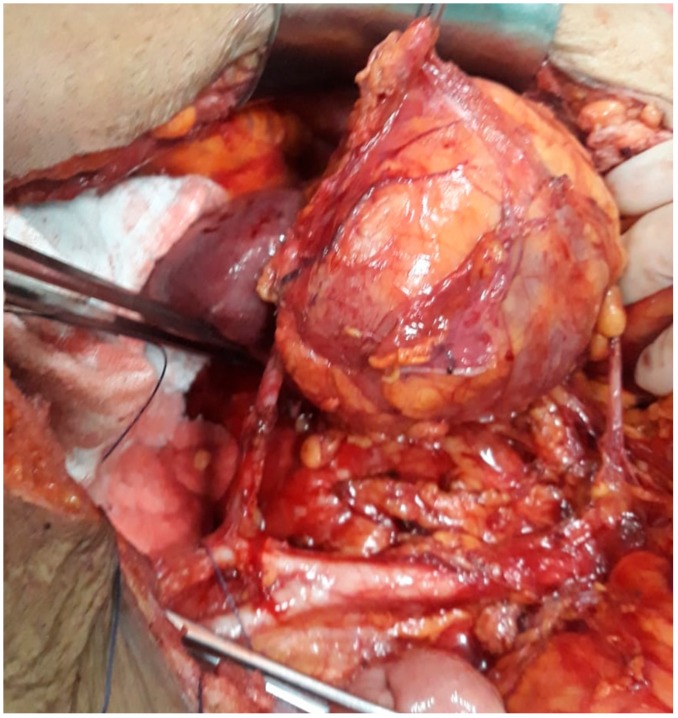
The final aspect after mobilization of the tumor en bloc with the left kidney and lymph node dissection.

This study was approved by the hospital ethical committee (the ethical code number was 93/21.8.2019). A 54-year-old postmenopausal female patient was investigated for diffuse pelvic pain, vaginal bleeding and fever, and was diagnosed with a large cervical tumor invading the rectum and the urinary bladder, in association with a massive peritumoral abscess. In the meantime, a left pelvic kidney, with no demarcation line with the tumoral process, was described during the preoperative computed tomography scan. At the time of presentation, the patient presented constant vesperal fever in association with biological inflammatory syndrome. Due to the finding of a large pelvic abscess in association with the clinical–biological condition of the patient, surgery as the first therapeutic intention was decided. Intraoperatively, a large pelvic tumor invading the rectum and the urinary bladder, in association with local perforation and a secondary abscess, was found. In the meantime, invasion of the upper renal pole was also certified (Figure 1, Figure 2, Figure 3 and Figure 4).

Therefore, total exenteration en bloc with a nephrectomy, as well as pelvic and para-aortic lymph node dissection was performed. The right ureter was exteriorized in a right terminal cutaneous ostomy, while the terminal end of the sigmoidian loop was exteriorized in a left cutaneous colostomy. The decision of not re-establishing the continuity of the digestive or urinary tract was taken due to the association of the tumoral perforation with a secondary pelvic abscess. The postoperative outcome was uneventful, the patient being discharged in the 14th postoperative day. The histopathological studies confirmed the presence of a moderately differentiated squamous cell carcinoma originating from the uterine cervix. At the one-month follow-up, the patient was referred to the oncology service in order to be submitted to adjuvant therapy and presented a satisfactory urinary function, with a mean level of creatinine of 1.2 mg/dL.

## 3. Discussion

Patients presenting ectopic kidneys with pelvic localization usually are at risk of developing hydronephrosis; therefore, in such cases, association of a pelvic malignancy can pose serious problems in terms of establishing whether the ureteral dilatation is induced by tumoral invasion or is the consequence of the pelvic disposition of the kidney [1,4]. So, clarification between the two situations should be carried out in order to correctly classify and stage the patient before deciding the therapeutic strategy [5,6]. In the case presented, the invasion was evident and affected the parenchymal area of the kidney and not the ureter, therefore a nephrectomy was needed. However, this circumstance of a pelvic kidney in association with cervical cancer has been rarely reported so far, with only a few case reports having been published. The most relevant ones are summarized in Table 1.

Interestingly, in the case reported by Ripley et al., the patient presented a pelvic kidney as the result of a previous kidney transplant; in this case, the radiation field could be established in a manner which avoided the renal involvement [7]. Similarly, in Abouna’s case report the patient also had been submitted nine years previously for a kidney transplantation, but in this case the therapeutic strategy when the cervical cancer was encountered was different and consisted of a renal replacement in the upper abdomen and revascularization by the use of a splenic artery followed by radiation therapy [8].

Another important issue that should be underlined in such cases is the one related to irradiation; patients presenting an association of a pelvic kidney and a locally advanced cervical tumor cannot be submitted to radiotherapy without risking injury to the kidney [9,10]; therefore, in such cases, including the kidney into the radiation field increases the risk of developing malignant hypertension and increases the chances to necessitate a therapeutic nephrectomy [9,10,11,12,13]. In such cases, certain authors proposed performing radical surgery followed by repositioning the kidney out of the pelvic area, in order to allow the patient to be further on submitted to the adjuvant treatment [14]. The first case of a patient presenting a pelvic kidney in association with cervical cancer, in whom the authors decided for a radical hysterectomy and kidney fixation in an extrapelvic area, followed by radiotherapy, was reported in 1980 by Rosenheim et al. [4]. However, in our case, due to the presence of a parenchymatous invasion of the kidney, nephrectomy was also imposed in order to achieve radical surgery.

Interestingly, in the case reported by Roth et al., published in 2004, both kidneys were found to have an ectopic location at the level of the pelvic area; moreover, the ureteral length was 9 cm in both ureters, making kidney fixation out of the pelvic area impossible, followed by radiation therapy. Therefore, the authors opted for per primam surgery, consisting of pelvic exenteration [5].

Recently, the American study group conducted by Lataifeh et al. reported the successful chemo-radiation of a patient with a stage IIB cervical tumor (IIB - Cervical carcinoma invades beyond the uterus, but not to the lower third of the vagina or to the pelvic wall with parametrial invasion) and ectopic kidney. The chemo-radiation protocol was applied with curative intent, with good oncological outcomes, but with the disadvantage of involving the pelvic kidney into the radiation field. However, the initial workup had revealed the fact that the left kidney was only partially functional; therefore, pelvic irradiation did not induce the development of renal insufficiency or malignant hypertension [6].

However, if surgery with radical intent is proposed, attention should be focused on the anatomical particularities of patients presenting a pelvic kidney [15,16]. Therefore, in such cases, the renal pedicle as well as the ureter can be situated in close contact with the iliac vessels, particular attention being needed in order to perform the pelvic lymph node dissection [17,18,19].

The single case series that has been published so far on this theme originates from India and included three such cases. The first case was initially diagnosed with a FIGO (International Federation of Obstetrics and Gynecology) stage IIB (Cervical carcinoma invades beyond the uterus, but not to the lower third of the vagina or to the pelvic wall with parametrial invasion) tumor and a right pelvic kidney and was submitted to external beam radiotherapy with a total dose of 500 Gy followed by a radical hysterectomy with bilateral adnexectomy and pelvic lymph node dissection, the ectopic kidney being successfully preserved. The second case was diagnosed with a stage IB1 cervical tumor and left pelvic kidney and was submitted to a total radical hysterectomy with bilateral adnexectomy and pelvic lymph node dissection. The third case was per primam submitted for a radical hysterectomy followed by adjuvant radiotherapy with a total dose of 500 Gy. In all cases, good oncological and renal long-term outcomes were obtained [2].

## 4. Conclusions

The association between pelvic kidneys and cervical cancer represents a scarce eventuality, only few cases being reported so far. In such patients, the therapeutic strategy should be carefully analyzed, with multiple dilemmas being reported so far. Moreover, the association between advanced cervical cancer and an ectopic kidney is even rarer, a single case which was finally submitted for anterior pelvic exenteration being reported so far. When it comes to the necessity of an association of total pelvic exenteration with a nephrectomy, to the best of our knowledge, this is the first case reported so far. The presence of a massive local invasion in the surrounding viscera, as well as renal invasion in association with the presence of a massive peritumoral abscess, enabled us to consider that total pelvic exenteration en bloc with a nephrectomy represent the best option for this case.

## Figures and Tables

**Table 1:** Relevant studies presenting a pelvic kidney in association with cervical cancer.

Name, Year	Age of the Patient (Years)	Presumed FIGO Stage—Preoperatively	Histopathological Type	Therapeutic Strategy	Follow-up
Bakri, 1993 [1]	65	IIB	Squamous cell carcinoma	Per primam surgery—radical abdominal hysterectomy en bloc with left parametrectomy, left ureteral resection and reimplantation into the urinary bladder using a Boari flap technique Followed by adjuvant chemotherapy—Cisplatinum 100 mg/m^2^ 3 weeks, 3 courses	Alive without recurrence at 6 years after surgery
Roth, 2003 [5]	48	IIB	Squamous cell carcinoma	Per primam surgery—anterior exenteration without vaginal reconstruction and distal ileal conduit	No evidence of disease at 14 months follow-up
Lataifeh,2007 [6]	50	IIB	Adenocarcinoma	Definitive radio-chemotherapy—4500 cGy and cisplatin with curative intent for 9 weeks	Disease free at two years follow-up, normal renal function
Ripley, 1995 [7]	NR	IB	Adenocarcinoma of the cervix in a previously kidney transplanted patient	Definitive external radiotherapy—4000 cGy and intracavitary radiotherapy with curative intent for 6.5 weeks	NR

FIGO, International Federation of Obstetrics and Gynecology; IIB, Cervical carcinoma invades beyond the uterus, but not to the lower third of the vagina or to the pelvic wall with parametrial invasion; IB, Invasive carcinoma with measured deepest invasion ≥5.0 mm, limited to the cervix uteri; NR, not reported.
